# Present documents on standardization of connection parts (interfaces and ports)

**DOI:** 10.1155/S1463924682000352

**Published:** 1982

**Authors:** U. Schmidt

**Affiliations:** C. H. F. Müller Aleanderstrasse 1 Posfach 10 46 40 Hamburg 1 2000 Germany


Journal of Automatic Chemistry, Volume 4, Number 3 (July–September 1982), pages 139–141

### Present documents on standardization of connection parts (interfaces and ports)

**U. Schmidt**

*C. H. F. Müller, Alexanderstrasse 1, Postfach 10 46 40, 2000 Hamburg 1, FR Germany*

An important problem with online data-processing in many analytical laboratories is the connection of different data-ports to analytical instruments. The use of standardized interfaces would go a long way towards solving this problem. This paper gives a status report on the efforts made towards reaching an internationally accepted standard for interfaces which can be used for devices in clinical laboratories.

The principal reasons that define the need for standardization are as follows. An analytical device delivered by supplier A generates data. These data are then processed by the laboratory data-processing system delivered by supplier B. This is schematically shown in figure 1.

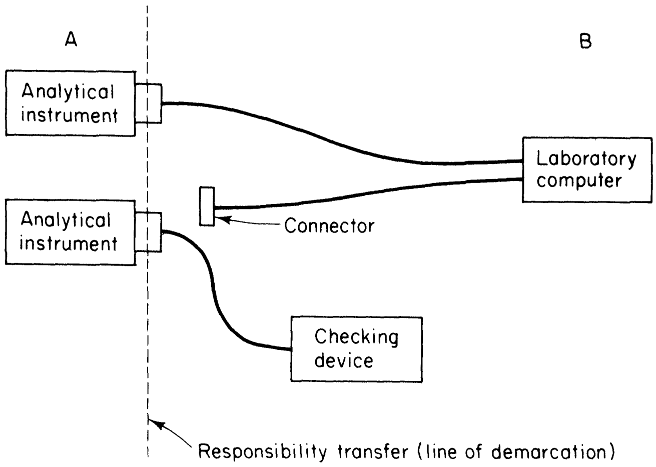

*Figure 1. General structure of laboratory systems.*

At some point in this configuration the responsibility for the data has to be transferred from supplier A to supplier B, and this point can be termed the 'line of demarcation'.

This demarcation aspect is of fundamental importance, particularly with regard to servicing the system. The user must have a guarantee that servicing and repair can be carried out without the responsibility for the fault being sent back and forth between the suppliers A and B. It is necessary, therefore, that an interface should have the following features:

(1) It should be well known and commonly used.
(2) It should be easy to check.
(3) The 'checking device' should be easily available (for example TELETYPE).
(4) The interface should be easy to connect to the checking device, so the connector should be positioned on the rear of the analytical instrument and should have a standard plug.

These items can be simply fulfilled if the interface itself is standardized between manufacturers and accepted internationally. In the past, with no accepted standard interfaces, many difficulties have arisen when users have attempted to connect analytical devices online to the laboratory computer. Historically, the development of digital interfaces for clinical analytical instruments started with printer interfaces, which were always tailored to the demands of the particular printer delivered by the manufacturer. Standardization of such constructions was almost impossible, and special interfaces had to be developed for each instrument, and for each application special software device handlers had to be written and tested.

Previous attempts to standardize interfaces for computer peripherals and analytical devices failed, primarily because of the competition between the different suppliers. To provide a standard interface outlet would allow the user to purchase the printer or computer from a competing company. Quite a different situation existed in the field of standardization of data-communication equipment interfaces. As a result of the influential role of the CCITT (Consultative Committee for International Telegraph and Telephone) there are international standards in this field, and these have become the basis for the construction of interfaces for the connection of peripheral devices to computers.

In 1975 a working group called 'Laboratory Data Processing' set up by the German Society for Medical Documentation, Informatics and Statistics (GMDS) in collaboration with numerous industrial companies, laid down specifications for hardware interfaces and basic data-link procedures for devices in clinical chemistry laboratories [1]. These specifications are a subset of the well-known CCITT V.24 and V.28 standards for data communication. All the essential information was defined in a document which had supplements relating to the analytical devices found in clinical laboratories.

In 1979 the GMDS specifications were incorporated into FR Germany's National Standards as DIN 66 258, Part 1 [2]. Fortunately the manufacturers of analytical instruments from other countries followed suit. For example, products from the USA are equipped with interfaces which are constructed according to US Standard RS232, which corresponds in most respects to V.24 and V.28.

An important reason for preparing the GMDS specifications was to collect in *one* document all the aspects of international standards already in use which were necessary to design an interface between an analytical instrument and a computer.

The contents of the GMDS paper can be described by the reference model shown in figure 2. This model was introduced in August 1979 by the International Standards Organization (ISO) for describing interfaces (ISO/TC97/SC16N227).

This reference model is very sophisticated, although it is used here in a simple manner. The model describes the functions of an interface in seven different layers. The following sections explain these layers, with reference to the GMDS specifications, by adding the appropriate definitions used in the GMDS paper.

### *Layer 1: the physical layer*

The physical layer provides mechanical and electrical functions. This layer is the basis of the reference model and defines the

0142 0453/82/0403 0139 $05·00 © 1982 Taylor & Francis Ltd

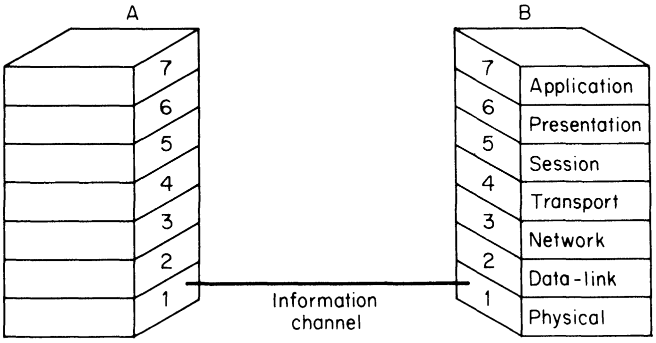

*Figure 2. ISO reference model for describing interfaces.*

transmission line, or channel, for transporting the bit-stream from A to B in figure 1. In the GMDS specifications two different interface versions are used: the V-S and C-S interfaces.

*V-S interface*

This interface works by voltage-controlled, bit-serial transmission.

(1) Electrical characteristics: voltage levels are in accordance with V.28, RS 232, and DIN 66 259, Part 1, respectively.
(2) Lines: lines are defined according to V.24, RS 232, and DIN 66 020, Part 1, respectively.
(3) Pin allocation is in accordance with ISO 2110, RS 232, and DIN 66 020, Part 1, respectively.

*C-S interface*

Since the V-S interface allows transmissions up to 30 m only, and the distances involved in clinical laboratories are generally greater than this, the C-S interface was introduced.

(1) Electrical characteristics: a 20 mA current loop is usually used; however, there is no standard for this.
(2) Lines—there is no existing standard.
(3) Pin allocation—again there is no existing standard.

As there are no standards for lines and pin allocation of a current loop interface, the following definitions were used in the GMDS paper:

| *Pin No.* | *Line* |
|---|---|
| 9: | transmitted data (plus polarity). |
| 10: | return, transmitted data. |
| 24: | received data (plus polarity). |
| 25: | return, received data. |

*V-S and C-S interface*

(1) Plug connector: a 25-pin connector, in accordance with IEC 48 B (CO) 96, and MIL-C-24308, respectively.
(2) Transmission mode asynchronous.
(3) Transfer rates: 110/1200/2400/9600 bit/s (in accordance with international standards).
(4) Character format: one start bit, seven information bits, one parity bit (even parity is in line with ISO 1177, and DIN 66 020, Part 1, respectively), and one or two stop bits.

### *Layer 2: data-link layer*

The data-link layer provides the procedures to implement the data transfer on layer 1. These procedures include certain checks, for example block checks. The two GMDS interfaces (V-S and C-S) use asynchronous basic mode procedures, partially in accordance with ISO 1745, ISO 2111, and DIN 66 019, respectively. The basic mode procedures need the control characters of a seven-bit code (see layer 6).

### *Layer 3: network layer*

The network layer provides the choice of information paths in the network, for example the dialled lines of a data-exchange system. This layer was not used in the present case.

### *Layer 4: transport layer*

The transport layer provides error checking of the data communication. For this purpose it receives a feedback from the procedure in layer 2. If the result in layer 2 is unsuccessful, then layer 4 has to decide what has to be done.

Currently the function of this layer must be implemented in the computer. Error checking is provided only as a special option of the GMDS interfaces and DIN 66 258, Part 1, respectively.

### *Layer 5: session layer*

The session layer decides who starts with a data transfer (A or B), and also controls the sending priority when using the dialogue mode. Transfers are possible without using this layer if the receiver is always in a ready condition and if the dialogue mode is not used.

### *Layer 6: presentation layer*

The presentation layer provides the presentation of data, i.e. coding, formatting, representation of numeric values, etc. The V-S and C-S interfaces are used for alphanumerics (seven-bit code according to ISO 646, CCITT Alphabet No. 5, ASCII, and DIN 66 003, respectively); and for numeric values (representation according to ISO/TC 97/SC 15 N 30, ECMA 7, and DIN 66 250, respectively).

### *Layer 7: application layer*

The application layer handles all application-dependent aspects. In an application in a clinical laboratory the contents of a data block, which is sent from the analytical device (side A) to the laboratory computer (side B), has to be defined. Within the computer a device-dependent handler has to be written to extract the relevant data.

### Future development

At the present time the International Standards Organization (ISO) is working on the modification of CCITT data transmission interface standards to data-processing peripheral interfaces. Thus work carried out in the past by national organizations working alone will now be co-ordinated by ISO.

The first ISO paper [3] has already been published. This paper includes the new CCITT electrical interface V.11 (equivalent to ANSI RS-423 and DIN 66 259 Part 2), which allows for data transmission over distances up to 1 km with data rates up to 9600 bit/s.

There are three major consequences of this action. Firstly in future there will be no layer 1 hardware problems. Secondly, with respect to layer 2, it remains to be decided which type of standardized procedure should be recommended. However, a considerable amount of work is required in layer 7. The contents of the data blocks required for transmission of test results in clinical laboratories must be fixed or at least generally described, for example the number of the analytical device, sample identification, method number, result, dilution, type of measurement, measuring range, error indication, status information, and so on.

This definition cannot be carried out by ISO independently, clinical chemists have a great deal of input into this. The author's view is that this work must be commenced immediately if the interfacing of analytical instruments and computers is to be carried out as a simple, routine procedure in the future.

### References

1. PORTH, A. J., KILLIAN, K. and PANGRITZ, H., *Hardware-Schnittstellen für den On-line-Anschluß von Geräten im klinisch-chemischen Labor an Datenverarbeitungsanlagen* (Deutsche Gesellschaft für Medizinische Dokumentation, Informatik und Statistik e.V. [GMDS], 1975).
2. Entwurf DIN 66 258 Teil 1: *Schnittstellen und Steuerungsverfahren für die Datenübermittlung* (Normenausschuß Informationsverarbeitung [NI] im DIN Deutsches Institut für Normung e.V., Beuth-Verlag, Berlin, 1979).
3. ISO TC97/SC6 N1961: *Data Terminal Equipment to Data Terminal Equipment (DTE to DTE) Interconnection.*

### Tectronica 83

Tectronica 83 will be held in June next year for four days and will show the latest developments in equipment and services for the life and physical sciences. The organizers, Industrial & Trade Fairs Ltd, intend that the show will be international and already have overseas representatives publicizing the event to manufacturers and visitors. Indeed, June 83 has been chosen partly because two major equipment shows: Analytica in Munich and Achema in Frankfurt are being held in 1982 and then not until 1984/85.

*Further information can be obtained from Industrial & Trade Fairs Ltd, Radcliffe House, Blenheim Court, Solihull, West Midlands B91 2BG, UK. Tel.: 021 705 6707.*

### Course announcement

*Computer systems for safety and control*

Oyez International Business Communications Ltd are organizing this course on behalf of the Technical Committee on Safety and Security of the European Workshop on Industrial Computer Systems. Computer systems are playing an increasingly important role in safety systems (for example shutdown and interlock systems for chemical and oil plants, steering computers for drilling rigs, shutdown systems for nuclear plants, elevator controllers, railway signalling systems, aircraft landing) their use poses serious problems, while at the same time offering a potential for much more advanced safety functions. The problems are of hardware and software unreliability. Oyez's two-day course presents an introduction to techniques which have been developed over the last 10 years for computer safety systems, and both practical examples and the latest research will be described. In order to make the course of especially practical value to participants, an in-depth study is made of a recently installed railway signalling system.

The following topics will be discussed: computer hardware for safety systems, software problems, software fault tolerance and redundancy programming, software debugging and testing, specification techniques for safety-related computer systems, measurement of software quality, software reliability assessment, systematic software verification, problems of concurrent programs, organization of safety-related computer projects, case studies and the potential for computers in safety.

'Computer systems for safety and control' will be held at the Portman Hotel, London on 22 and 23 September 1982.

*Further details from Leslie Claff, Oyez IBC Ltd, Norwich House, 11–13 Norwich Street, London EC4A 1AB. Tel.: 01 242 2481.*

